# A reflection on ‘Bimetallic mechanism operating in the copolymerization of propylene oxide with carbon dioxide catalyzed by cobalt–salen complexes’

**DOI:** 10.1039/d5sc90244d

**Published:** 2025-11-11

**Authors:** Koji Nakano, Kyoko Nozaki

**Affiliations:** a Department of Applied Chemistry, Graduate School of Engineering, Tokyo University of Agriculture and Technology Koganei 184-8588 Japan k_nakano@cc.tuat.ac.jp; b Department of Chemistry and Biotechnology, Graduate School of Engineering, The University of Tokyo Tokyo 113-8656 Japan nozaki@chembio.t.u-tokyo.ac.jp

## Abstract

The development of catalysts that exploits cooperative interactions between two distinct metal centers has emerged as a powerful strategy in modern organic transformations. In 2010, we reported dinuclear cobalt complexes for the alternating copolymerization of epoxides with carbon dioxide, in which an intramolecular bimetallic propagation mechanism operates to afford high catalytic activity (K. Nakano, S. Hashimoto, and K. Nozaki, *Chem. Sci.* 2010, **1**, 369–373, https://doi.org/10.1039/C0SC00220H). In this reflection, we provide a brief overview of subsequent studies on cooperative bimetallic catalysts for the epoxide/CO_2_ copolymerization.

## Abstract 2

In celebration of our 15th anniversary and some of our most popular articles, Koji Nakano and Kyoko Nozaki reflect on the area of bimetallic polymerisation catalysts, building on the first paper published in *Chemical Science* on this topic: https://doi.org/10.1039/C0SC00220H.

## Graphical abstract

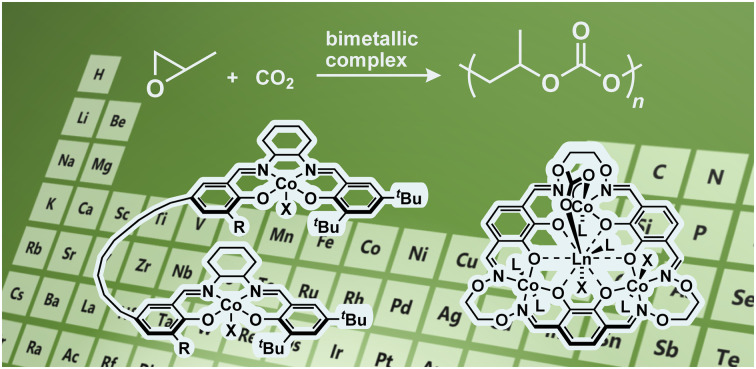

## 1. Introduction

**Fig. 1:**
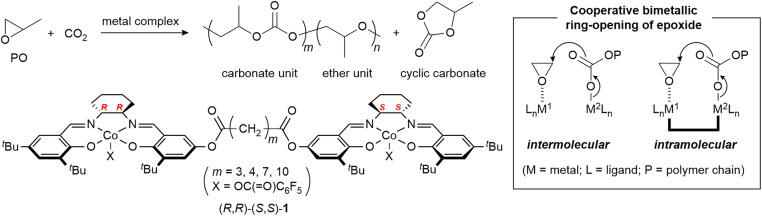
Alternating copolymerization of PO with CO_2_ with dinuclear (salen)Co complexes.

Over half a century has passed since the first report on the alternating copolymerization of epoxides with carbon dioxide (CO_2_) by Inoue and coworkers.^1^ This copolymerization has long been a subject of research as it allows the transformation of CO_2_—an abundant, renewable, and intrinsically non-toxic carbon feedstock—into value-added aliphatic polycarbonates. Following the first report using a Et_2_Zn/water catalyst system, a wide range of heterogeneous and homogeneous metal-based catalysts have been designed, leading to significant advances in catalytic performance. In 2010, our article published in *Chemical Science* (https://doi.org/10.1039/C0SC00220H) reported the dinuclear (salen)Co complexes 1, demonstrating higher catalytic activity than mononuclear (salen)Co complexes which were reported as highly active catalysts for the copolymerization (basic skeleton of salenH_2_ = *N*,*N*′-disalicylidene-1,2-ethylenediamine) (Fig. 1).^2^ Our catalyst design is based on the proximity effect in a cooperative bimetallic propagation mechanism: (a) one metal center activates the epoxide through coordination and (b) the other delivers the carbonate propagating species to the activated epoxide. In the following sections, we briefly summarize advances in catalyst design in light of bimetallic propagation mechanisms, including some seminal examples prior to our 2010 study.

## 2. Homobimetallic complexes

**Fig. 2:**
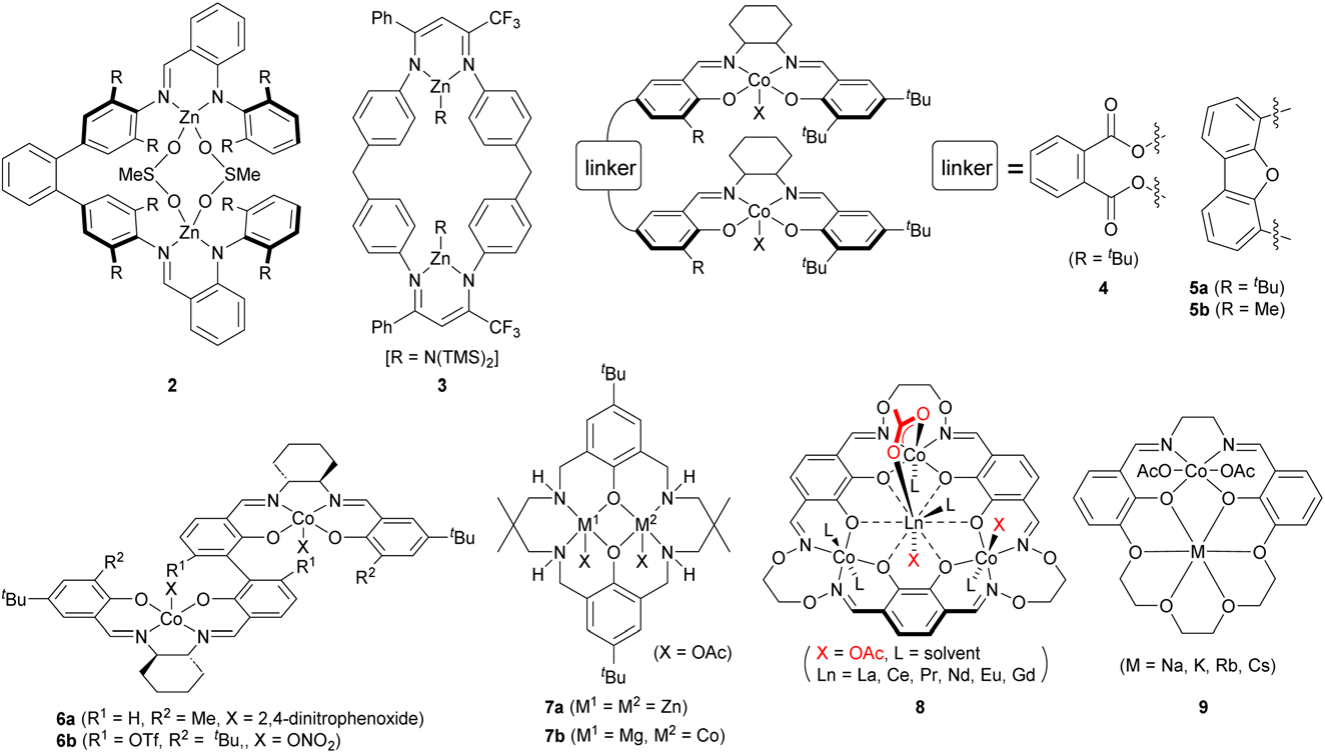
Di- and multinuclear metal complexes for the epoxide/CO_2_ copolymerization.

Since the mid-1990s, well-defined homogeneous metal-based catalysts have emerged, achieving great progress in catalytic activity and selectivity (polymer/cyclic, regio- and stereochemistry, *etc.*). In 1998, Coates and coworkers reported a series of zinc complexes with β-diiminate (BDI) ligands, which exhibit high activity for the cyclohexene oxide (CHO)/CO_2_ copolymerization (TOF: up to 4980 h^−1^).^3,4^ Kinetic studies with a series of (BDI)Zn complexes demonstrated 1.0–1.8 order dependence in total zinc concentration, indicating an intermolecular cooperative bimetallic mechanism is in operation.^5^ This mechanistic insight has led to the development of several dizinc complexes, such as 2 and 3 (Fig. 2), in which two (BDI)Zn units are incorporated into a single molecule.^6–9^ Taking advantage of proximity effects, significantly high TOF can be achieved (up to 155 500 h^−1^) for the CHO/CO_2_ copolymerization even under highly diluted conditions.

(Salen)metal complexes are the most investigated homogeneous catalysts for the epoxide/CO_2_ copolymerization. In 2003, Coates and coworkers reported the pioneering application of (salen)Co complexes.^10^ Similar to the ring-opening mechanism in (salen)Co-catalyzed asymmetric hydrolysis of epoxides, we anticipated that the ring-opening step in the epoxide/CO_2_ copolymerization can proceed *via* a cooperative bimetallic mechanism. Accordingly, in our 2010 study,^2^ we designed a series of dinuclear (salen)Co complexes 1 which can enforce the intramolecular bimetallic propagation mechanism. The propylene oxide (PO)/CO_2_ copolymerization with 1 affords poly(propylene carbonate) (PPC) with an ether linkage to some extent (carbonate/ether: 97/3–82/18). Interestingly, the relative configuration of the two salen ligands has great impact on catalytic activity. The heterochiral (*R*,*R*)–(*S*,*S*)-1 exhibits three- to five-fold higher activity than the homochiral (*R*,*R*)–(*R*,*R*)-1. The alkylene linker length also affects the catalytic performance, and (*R*,*R*)–(*S*,*S*)-1 with a butane-1,4-diyl linker is most active (up to 430 h^−1^). The most active (*R*,*R*)–(*S*,*S*)-1 exhibits the lowest carbonate/ether selectivity among the dinuclear complexes examined. The dinuclear complexes 1 can keep their catalytic activity under highly diluted conditions owing to proximity effects, while a mononuclear (salen)Co complex exhibits much lower activity. All of these results strongly support the intramolecular cooperative bimetallic mechanism operating in the copolymerization. Addition of onium salts, such as [Ph_3_P=N=PPh_3_]Cl ([PPN]Cl), to a mononuclear (salen)Co complex (1.0 equiv. to Co) has been reported to enhance the catalytic activity at ambient temperature,^11,12^ while the copolymerization at higher temperature resulted in cyclic carbonate formation. In our 2010 study, the use of 0.5 equiv. of [PPN]Cl improved catalytic activity (TOF >1700 h^−1^) while maintaining high polymer/cyclic selectivity. The activities observed with mono- and dinuclear Co complexes are comparable, indicating the monometallic mechanism operates in the presence of onium salts.

One of the authors, Nakano, employed the same approach in his follow-up publications, in which two (salen)Co units are linked by more rigid linkers with the expectation that the rigid linker enforces a geometry between the two Co centers more appropriate for the epoxide ring-opening step compared to the flexible alkylene linker used in our 2010 study. First, Nakano investigated the use of a phenylene linker.^13^ The dinuclear (salen)Co complexes 4 with a 1,2-phenylene linker exhibits higher catalytic activity and lower carbonate/ether selectivity than the 1,3-phenylene-linked analogue. As observed for the alkylene-linked dinuclear (salen)Co complexes, the dicobalt complex (*R*,*R*)–(*S*,*S*)-4 with a heterochiral combination, demonstrated much higher activity (up to 182 h^−1^) than the homochiral analogue. In addition, the heterochiral complex gives lower carbonate/ether selectivity. Thus, the heterochiral combination is presumed to position two (salen)Co units in closer proximity than the homochiral one. The phenylene-linked complex (*R*,*R*)–(*S*,*S*)-4 exhibits slightly higher TOF than the alkylene-linked complex (*R*,*R*)–(*S*,*S*)-1 at highly diluted conditions of [PO]/[Co] = 10 000.

Nakano and coworkers also designed complex 5, where the (salen)Co units are directly incorporated onto a rigid dibenzofuran backbone to induce a more restricted conformation than 1 and 4.^14^ The heterochiral complex again gave much higher TOF than the homochiral one. The sterically less bulky methyl substituent at the 6 position of the salicylaldimine unit, is favorable for higher catalytic activity. The most active complex (*R*,*R*)–(*S*,*S*)-5b achieves a TOF of up to 248 h^−1^. Under the same polymerization conditions, complexes (*R*,*R*)–(*S*,*S*)-1, 4, and 5b displayed comparable activity. The complex (*R*,*R*)–(*S*,*S*)-5b can be applied to the copolymerization with 1-hexene oxide, whereas the complex (*R*,*R*)–(*S*,*S*)-4 cannot produce the copolymer with 1-butene oxide.

Following our publication in 2010 in *Chemical Science*, a wave of related research efforts have been inspired by the catalyst design of linking two mononuclear metal complexes active in the epoxide/CO_2_ copolymerization.^15^ For instance, dinuclear (salphen)Cr complexes^16,17^ and (amino-triphenolato)Fe complexes,^18^ have been developed and were found to be active at very low catalyst loadings in the PO/CO_2_ or CHO/CO_2_ copolymerization. In 2013, Lu and coworkers introduced biphenyl-linked dinuclear (salen)Co complex 6a for the asymmetric copolymerization of *meso*-epoxides with CO_2_.^19^ The binaphthyl-linked analogue has been reported to catalyze highly enantiomer-selective homopolymerization of terminal epoxides,^20^ while it shows low activity and enantiomer-selectivity in the epoxide/CO_2_ copolymerization. The biphenyl bridge with a certain degree of flexibility, offers an inside cleft suitable for intramolecular cooperative bimetallic ring-opening, leading to unprecedented activity and enantioselectivity (up to >99% ee).^21^ A wide range of *meso*-epoxides can be copolymerized with complexes 6a, and the resulting highly stereoregular polycarbonates were found to be semicrystalline.^22–24^ Very recently, the same group has computationally designed novel dinuclear (salen)Co complexes 6b, achieving highly efficient kinetic-resolution copolymerization of racemic terminal epoxides with CO_2_.^25^

In 2009, Williams and coworkers introduced the dinuclear zinc complex with a diphenolate tetraamine macrocyclic ligand.^26^ The macrocyclic ligand is advantageous in that the two metal centers can be precisely arranged to promote an intramolecular cooperative bimetallic mechanism. This catalyst design should be distinguished with the one linking two mononuclear metal complexes described above. It is noteworthy that the dizinc complex 7a works at just 1 atm of CO_2_ with a TOF of 25 h^−1^, affording a completely alternating main-chain sequence. The same group applied the macrocyclic ligand to dicobalt and dimagnesium complexes, achieving a higher TOF at 1 atm of CO_2_ (up to 500 h^−1^).^27,28^

## 3. Heterobimetallic complexes

It is reasonable to assume that different metals would be optimal for the two distinct roles—one metal activates an epoxide, and the other delivers a propagating carbonate chain end—in a cooperative bimetallic mechanism. Therefore, a heterometallic system is the more promising catalyst design. Since 2014, Williams and colleagues have expanded their successful investigation on homometallic dinuclear complexes to heterometallic analogues.^29–31^ Among them, the heterodinuclear Mg–Co complex 7b exhibits the highest TOF of 1205 h^−1^ under 1 bar of CO_2_ and 12 460 h^−1^ under 20 bar of CO_2_. Kinetic analyses imply the cooperative interaction between the metals, in which the Mg center activates the epoxide through coordination, and the Co center provides the reactive (nucleophilic) carbonate chain end.

In 2020, one of the authors, Nozaki developed Co_3_–Ln heterobimetallic tetranuclear complexes 8.^32^ Each complex catalyzes the CHO/CO_2_ copolymerization, yielding the alternating copolymer with little cyclic carbonate by-product. The Nd-incorporated complex 8 demonstrated the highest TON of 13 000, one of the highest values ever reported for multimetallic systems, and TOF of 1625 h^−1^. The copolymerization is proposed to proceed *via* a monomolecular bimetallic mechanism: CHO is activated by the Ln ion and attacked intramolecularly by the propagating carbonate chain end on the Co center. Within the same year, Williams and coworkers have employed a novel macrocyclic ligand, which combines a salen unit with a crown-ether unit, to prepare a series of heterometallic Co–alkali metal heterobimetallic dinuclear complexes 9.^33,34^ The best catalyst is the Co–Na complex, showing a TOF of 1590 h^−1^ under 1 bar of CO_2_ for the CHO/CO_2_ copolymerization. It is noteworthy that the Co–Na complex 9 can also copolymerize PO with CO_2_ with high activity of 800 h^−1^ (20 bar CO_2_).

## 4. Outlook

Our 2010 study significantly reinforced catalyst design, integrating cooperative bimetallic propagation mechanisms and proximity effects. This catalyst design has been widely adopted and has contributed to the development of high-performance epoxide/CO_2_ copolymerization catalysts. Particularly, heterometallic systems, in which each metal center fulfils its optimal role, represent the leading candidates. There remains, however, scope for further investigation on di- or multinuclear metal catalysts, including the selection and combination of metals and ligand design. In these future investigations, catalyst design based on mechanistic studies employing experimental and theoretical techniques would continue to be of critical importance. Although beyond the scope of the present reflection, another major line of catalyst design is “two-components in one molecule”,^35^ such as (salen)Co complexes and organoboranes tethering onium salts. In these catalyst systems, a Lewis acidic unit activates an epoxide, and an onium unit delivers a propagating chain end intramolecularly. Accordingly, the principles of catalyst design are essentially the same as those based on dinuclear metal complexes. Advances in catalyst systems has intensified global efforts toward the commercialization of CO_2_-based polycarbonates. Continuous progress in catalyst development is key to enabling the practical application of epoxide/CO_2_ copolymerization technology.

## Conflicts of interest

There are no conflicts to declare.
